# Supplementary figures and images for: Drosophila mechanical nociceptors preferentially sense localized poking
Source: eLife. 2022 Oct 6;11:e76574. doi: 10.7554/eLife.76574 (PMC9678358; doi:10.7554/eLife.76574)

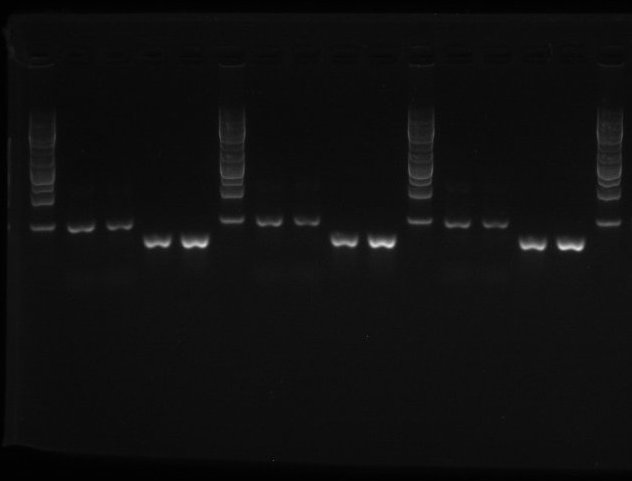

Supplement: Figure 6—figure supplement 2—source data 1. [file elife-76574-fig6-figsupp2-data1.zip › Figure 6-figure supplement 2-soure data 1/Figure 6-figure supplement 2_source data 1_raw image of gel.jpg]
